# Characterization and Genetic Variation of *Vibrio cholerae* Isolated from Clinical and Environmental Sources in Thailand

**DOI:** 10.1371/journal.pone.0169324

**Published:** 2017-01-19

**Authors:** Achiraya Siriphap, Pimlapas Leekitcharoenphon, Rolf S. Kaas, Chonchanok Theethakaew, Frank M. Aarestrup, Orasa Sutheinkul, Rene S. Hendriksen

**Affiliations:** 1 Department of Microbiology, Faculty of Public Health, Mahidol University, Bangkok, Thailand; 2 National Food Institute, Technical University of Denmark, Research Group for Genomic Epidemiology, WHO Collaborating Center for Antimicrobial Resistance in Foodborne Pathogens and Genomics and European Union Reference Laboratory for Antimicrobial Resistance, Kgs. Lyngby, Denmark; 3 Faculty of Public Health, Thammasat University, Rangsit Center, Pathumthani, Thailand; Midwestern University, UNITED STATES

## Abstract

Cholera is still an important public health problem in several countries, including Thailand. In this study, a collection of clinical and environmental *V*. *cholerae* serogroup O1, O139, and non-O1/non-O139 strains originating from Thailand (1983 to 2013) was characterized to determine phenotypic and genotypic traits and to investigate the genetic relatedness. Using a combination of conventional methods and whole genome sequencing (WGS), 78 *V*. *cholerae* strains were identified. WGS was used to determine the serogroup, biotype, virulence, mobile genetic elements, and antimicrobial resistance genes using online bioinformatics tools. In addition, phenotypic antimicrobial resistance was determined by the minimal inhibitory concentration (MIC) test. The 78 *V*. *cholerae* strains belonged to the following serogroups O1: (n = 44), O139 (n = 16) and non-O1/non-O139 (n = 18). Interestingly, we found that the typical El Tor O1 strains were the major cause of clinical cholera during 1983–2000 with two Classical O1 strains detected in 2000. In 2004–2010, the El Tor variant strains revealed genotypes of the Classical biotype possessing either only *ctxB* or both *ctxB* and *rstR* while they harbored *tcpA* of the El Tor biotype. Thirty O1 and eleven O139 clinical strains carried CTXϕ (Cholera toxin) and *tcpA* as well four different pathogenic islands (PAIs). Beside non-O1/non-O139, the O1 environmental strains also presented *chxA* and Type Three Secretion System (TTSS). The *in silico* MultiLocus Sequence Typing (MLST) discriminated the O1 and O139 clinical strains from other serogroups and environmental strains. ST69 was dominant in the clinical strains belonging to the 7^th^ pandemic clone. Non-O1/non-O139 and environmental strains showed various novel STs indicating genetic variation. Multidrug-resistant (MDR) strains were observed and conferred resistance to ampicillin, azithromycin, nalidixic acid, sulfamethoxazole, tetracycline, and trimethoprim and harboured variants of the SXT elements.

For the first time since 1986, the presence of *V*. *cholerae* O1 Classical was reported causing cholera outbreaks in Thailand. In addition, we found that *V*. *cholerae* O1 El Tor variant and O139 were pre-dominating the pathogenic strains in Thailand. Using WGS and bioinformatic tools to analyze both historical and contemporary *V*. *cholerae* circulating in Thailand provided a more detailed understanding of the *V*. *cholerae* epidemiology, which ultimately could be applied for control measures and management of cholera in Thailand.

## Introduction

*Vibrio cholerae* is the causative agent of the severe, watery diarrheal disease cholera. *V*. *cholerae* is classified into approximately 206 serogroups of which O1 and O139 have the potential to cause cholera outbreaks and are associated with cholera pandemics. The remaining serogroups; determined non-O1/non-O139 are often referred to as environmental cholera [1–3] and part of the normal flora of aquatic ecosystems [4]. Nonetheless, some non-O1/non-O139 strains have the potential to cause mild diarrhea, and outbreaks have been observed in several countries including Thailand [5–7]. The serogroup O1 is divided into two biotypes: Classical and El Tor, based on phenotypic differences [2].

Since 1817, cholera has spread from the Indian sub-continent and seven pandemics have been observed, the seventh of which is still ongoing. The first six pandemics were associated with the O1 Classical biotype and ceased around 1923 [8, 9]. In 1961, the 7^th^ pandemic began in Southeast Asia, caused by the O1 El Tor biotype [3, 10–13]. Whole genome sequence (WGS) analysis has identified eight distinct phylogenetic lineages: L1-L8 with L1 and L3-L6 representing the former pandemics and L2 the present 7^th^ El Tor pandemic. Lineages L7 and L8 are formed by unique isolates [12]. The lineage L2 of the 7^th^ pandemic has further been subdivided into three waves; I, II and III, of which, wave III seems to consist of several clusters [3, 12]. In general, the clusters separate isolates from Africa and India from those isolated in Haiti, Nepal, and Southeast Asia [12, 14]. In 1992, *V*. *cholerae* O139 emerged and caused epidemic cholera [15] followed in 2002 by the emergence of *V*. *cholerae* O1 variants; a genetic mixture of the Classical and El Tor biotypes. The *V*. *cholerae* O1 variants were later reported in several countries in Africa and Asia [16–19]. Since 2013, after the containment of the cholera outbreak in Haiti, the number of reported cholera cases has decreased globally. In Asia however, the incidence of cholera has increased and continues to pose a serious public health concern [20].

*V*. *cholerae* consists of two chromosomes and the hallmark of pathogenic *V*. *cholerae* is the major virulence factors; cholera toxin (CT) and toxin co-regulated pilus (TCP). The two virulence factors are clustered within two regions; the *Vibrio* pathogenicity island I (VPI-1) encoded by TCP [21] and the CTX genetic element comprised by a core region in CTXϕ. The latter contains not only the genes of the cholera toxin, *ctxAB*, but also carries the zonular occludens toxin (*zot*) and accessory colonization enterotoxin (*ace*) [22]. In addition, other virulence genes encoding hemolysin (*hlyA*), heat stable enterotoxin (*stn*), mannose-sensitive hemagglutin pilus (*mshA*), repeats-in-toxin A toxin (*rtxA*), and a ToxR regulatory protein (*toxR*) have been associated with diarrheal disease [23, 24]. Recently, the type III secretion system (TTSS) has been known as a key virulence factor and appears to be an important virulence factor for pathogenicity of non-O1/non-O139 [25].

Since 1997, endemic or sporadic cholera cases have been linked every year to contaminated seafood or potable water in Thailand [26]. Antimicrobial treatments have been recommended for only severe dehydration cases. Nonetheless, the occurrence of resistant strains has dramatically increased [27]. The presence of the SXT element and class I integron have been reported to contribute to the spread of antimicrobial resistance genes among *V*. *cholerae* and other bacteria [28].

The objective of this study was to provide more knowledge of the genotypic variation in *V*. *cholerae* observed during the past three decades in Thailand. A collection of clinical and environmental *V*. *cholerae* serogroup O1, O139, and non-O1/non-O139 strains collected between 1983 and 2013 in Thailand were characterized by a combination of conventional microbiological tests, molecular methods, next generation sequencing, and bioinformatics tools to determine the pheno- and genotypes. In addition, the distribution of virulence-associated genes and the occurrence of antimicrobial resistance and corresponding resistance genes including the class 1 integron and SXT element among *V*. *cholerae* strains were subsequently analyzed to elucidate the emerging antimicrobial resistance and virulence properties.

## Materials and Methods

### Bacterial strains

A total of 78 *V*. *cholerae* strains were selected for this study based on the serogroups O1, O139, and non-O1/non-O139, the sources for these strains were the clinic and environment, and date (1983–2013) from the culture collection of the Department of Microbiology, Faculty of Public Health, Mahidol University, Thailand (Table A in S1 File). The clinical strains were previously isolated from stools and rectal swabs of patients suffering from sporadic cases or outbreaks of cholera in central Thailand and the environmental strains were isolated from seafood, water, and hand swabs.

### Characterization of *V*. *cholerae*

The purity of all *V*. *cholerae* strains were assessed on Thiosulfate-citrate-bile salts-sucrose (TCBS) agar prior to confirmation using a combination of biochemical, serological, and molecular methods as previously described [29, 30]. Serogroups and serotypes were determined by slide agglutination utilizing specific polyvalent antisera against *V*. *cholerae* O1 and O139, and monovalent specific to Inaba and Ogawa antisera (S & A Reagents Lab, Bangkok, Thailand) and by touchdown-multiplex polymerase chain reaction (TMPCR) using species-specific primers for *V*. *cholerae* (*ompW* gene) and serogroup-specific for O1 (*rfbV* gene) and O139 (*wbfZ* gene) [30].

All *V*. *cholerae* O1 strains were classified according to biotypes using the quality control strains; O395 (O1 Classical), N16961 (O1 El Tor), and MO45 (O139) and based on the combination of previously described conventional biotyping methods [31] and genotypically by a bioinformatics tool: MyDbFinder (https://cge.cbs.dtu.dk/services/MyDbFinder/) as previously described [32].

### Antimicrobial susceptibility testing

Antimicrobial susceptibility to ampicillin (AMP), azithromycin (AZM), cefotaxime (CTX), chloramphenicol (CHL), ciprofloxacin (CIP), gentamicin (GEN), meropenem (MEM), nalidixic acid (NAL), sulfamethoxazole (SMX), ceftazidime (CAZ), tetracycline (TET), tigecycline (TGC), and trimethoprim (TMP) was performed by broth microdilution to determine minimum inhibitory concentration (MIC) with a commercially prepared, panel of dehydrated antimicrobials (Sensititre; TREK Diagnostic Systems Ltd., East Grinstead, England). Antimicrobial susceptibility test results were interpreted according to Clinical and Laboratory Standards Institute (CLSI) breakpoints [33], except for tigecycline, for which the clinical breakpoint was used according to the European Committee on Antimicrobial Susceptibility Testing (EUCAST) recommendations (http://www.eucast.org). *Escherichia coli* ATCC 25922 was used as reference strain for quality control according to CLSI guidelines [33].

### Whole genome sequencing

*V*. *choleare* genomic DNA was extracted using the Invitrogen Easy-DNA^TM^ Kit (Invitrogen, Carlsbad, CA, USA). The concentrations of the extracted DNA were determined using a Qubit dsDNA BR assay kit (Invitrogen). The genomic DNA was prepared for Illumina paired-end sequencing using the Illumina (Illumina, Inc., San Diego, CA) NexteraXT® Guide 150319425031942 following protocol revision C. A sample of pooled NexteraXT Libraries was loaded onto an Illumina MiSeq reagent cartridge using MiSeq Reagent Kit v2 and 500 cycles with a Standard Flow Cell. The libraries were sequenced using the MiSeq Illumina platform and MiSeq Control Software 2.3.0.3. All strains were paired-end sequenced.

Raw sequence data were submitted to the European Nucleotide Archive (http://www.ebi.ac.uk/ena) under study accession no.: PRJEB14630 (http://www.ebi.ac.uk/ena/data/view/PRJEB14630). The raw reads were assembled using the Assemble pipeline (version 1.0) available from the Center for Genomic Epidemiology (CGE; http://cge.cbs.dtu.dk/services/all.php) based on the Velvet algorithms for *de novo* short reads assembly. A complete list of genomic sequence data is available in Table B in S1 File.

### The use of bioinformatics tools

#### Identification of *V*. *cholerae* and determination of associated virulence genes and pathogenicity islands

MyDbFinder is a BLAST-based search-engine that was developed as “an empty database” in the same format as the ResFinder tool [34] to identify user-defined genes (https://cge.cbs.dtu.dk/services/MyDbFinder/). The users populate their own database by including DNA sequences of interest in FASTA format into a pure text file. MyDbFinder query raw reads or assembled genome data and outputs the best matching genes from the user’s database.

The web-server MyDbFinder 1.0 was used to, *in silico*, determine the species-specific gene (*ompW*), serogroup-specific genes (*rfbV*-O1, *wbfZ*-O139), biotypes-specific genes (*ctxB*, *rstR*, *tcpA*), specific gene (VC2346) of the 7^th^ pandemic strain, putative virulence genes (*ctxA*, *ctxB*, *zot*, *ace*, *tcpA*, *hlyA*, *stn*, *chxA*, *rtxA*, *ompU*, *toxR*, *mshA*, TTSS), and pathogenic islands (PAI): (VPI-1, VPI-2, VSP-1, VSP-2) in all *V*. *cholerae* strains with a selected threshold equal to 95% identity as previously described [32]. The genes used in this study are shown in Table C in S1 File.

#### Determination of antimicrobial resistance genes, SXT element, and class 1 integron

In all *V*. *cholerae* strains, antimicrobial resistance genes were detected based on the assembled sequences using the ResFinder tool (version 2.1, 80% threshold for %ID/ 60% minimum length) available from CGE [34]. The SXT element, class 1 integron, and presence of mutation in the DNA gyrase gene (*gyr*A), and the DNA topoisomerase IV genes (*par*C and *par*E) were determined using MyDbFinder as previously described [32]. The nucleotide sequence of integrase gene of the SXT element (*int*_SXT_), the class 1 integron (*int*I), *gyr*A, *par*C, and *par*E genes of the quinolone-resistant *V*. *cholerae* strains from GenBank were used as references (Table C in S1 File).

ICE*Vc*Hai1 (JN648379) and *dfr*A18 gene of SXT^MO10^ (AY034138) were used as templates in MyDBFinder (threshold, 95% identity) to determine which *V*. *cholerae* strains contained an *int*_SXT_ gene.

#### Multilocus sequence type

The assembled sequences were analyzed to identify the MLST, sequence type (ST) for *V*. *cholerae* using the MLST tool (version 1.7) available from CGE [35]. The seven housekeeping genes: *adk*, *gyrB*, *metE*, *mdh*, *pntA*, *purM*, and *pyrC* as previously described by Octavia *et al*. (2013) [36], were extracted from 78 *V*. *cholerae* genomes in this study and 6 *V*. *cholerae* genomes from the NCBI database (M66-2, O395, N16961, MO45, MS6, 2010EL-1786). Concatenation of the housekeeping gene sequences was performed with an in-house python script. A multiple alignment was created from the concatenated sequences using MUSCLE via MEGA5 [37]. The final phylogenetic MLST tree was constructed by MEGA5 using the maximum likelihood method of 1,000 bootstrap replicates using Tamura-Nei model for inference [38]. Figtree (http://tree.bio.ed.ac.uk/software/figtree/) was used to visualize the tree. The confidence of the nodes in the tree is estimated by bootstrap values, calculated by sampling with replacements from the multiple sequence alignment. New STs were confirmed by PCR as previously described Octavia *et al*. (2013) [36].

#### Genomic islands in the chromosomes of *V*. *cholerae*

Variation of genomic islands including CTX, VPI-1, VPI-2, VSP-1, VSP-2, and the super-integron were visualized and determined based on chromosome I and II of the reference genome *V*. *cholerae* N16961 (accession no. AE003852 and AE003853) using a BLAST atlas. All protein sequences from the reference genome were aligned against other *V*. *cholerae* genomes using BLASTP. The presence and absence of genes were visualized in a circle, with greater similarity represented by higher intensity of color [39].

## Results

### Characterization of *V*. *cholerae* strains

**Fig 1:**
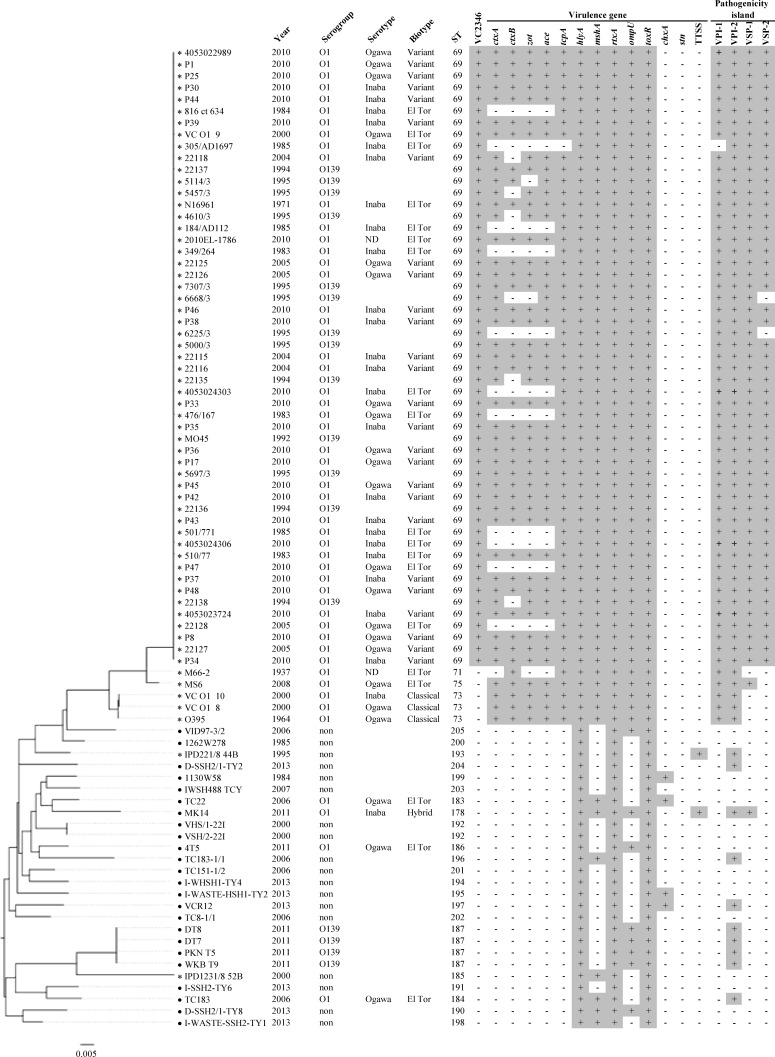
*In silico* MLST tree of *Vibrio cholerae* strains related with virulence gene profiles. The *V*. *cholerae* clinical (*) and environmental (•) strains in Thailand were related to pandemic and epidemic strains. Seven housekeeping genes were extracted from *V*. *cholerae* genomes. The phylogenetic tree was generated by FigTree.

Of the 78 *V*. *cholerae* strains investigated, 44 belonged to serogroup O1, 16 to O139, and 18 to non-O139/non-O1. Among the 44 *V*. *cholerae* O1 strains, 24 strains were identified as Inaba and 20 strains as Ogawa (Fig 1, Table D in S1 File).

The biotype classification of the 44 *V*. *cholerae* O1 strains revealed 15 strains determined as being typical El Tor similar to the phenotype of El Tor strain N16961 (CCA^+^ HSE^+^ PB^r^ VP^+^). The 15 strains all carried according to MyDbFinder identical genes; *ctxB*, *rstR*, and *tcpA* with the exception of three environmental strains (TC22, MK14, and 4T5) and one clinical strain (TC183). Two strains (VC O1-8 and VC O1-10) belonged to the biotype Classical, exhibiting the phenotype CCA^-^ HSE^-^ PB^s^ VP^-^ and genotypically similar to O395 strain (Classical). Furthermore, 26 *V*. *cholerae* O1 strains tested phenotypically El Tor but revealed using MyDbFinder mixed Classical and El Tor genotypes and determined as an El Tor variant. Finally, one *V*. *cholerae* O1 strain (MK14) expressed phenotypically both biotypes (CCA^+^ HSE^+^ PB^s^ VP^+^) and was determined as belonging to the hybrid biotype (Fig 1, Table D in S1 File).

The MLST types of the 78 *V*. *cholerae* and 6 reference genomes were analyzed and assigned to 26 different STs (Fig 1). The analysis showed that 50 strains were represented by ST69, making this the most common ST and all 50 of these strains related to clinical strains. Among clinical strains, 38 O1 El Tor and 12 serogroup O139 belonged to the same cluster with the pandemic strains (N16961 and MO45) and the Haitian strain (2010EL1786). The strains harbored the 7^th^ pandemic-specific gene (VC2346) according to MyDbFinder, suggesting that they belong to the same clonal linage. The cluster is also linked to the pre-6^th^ pandemic strain (M66-2) and the endemic strain from Thailand (MS6), which was closely related to the cluster of the O1 Classical strains (ST73) including the strains related to the 6^th^ pandemic (Table E in S1 File). All of the non-O1/non-O139 strains and the environmental strains, except for four O139 strains belonging to ST187, were assigned to different novel STs, suggesting a high degree of genetic diversity.

### Distribution of virulence-associated genes and pathogenicity islands

**Fig 2:**
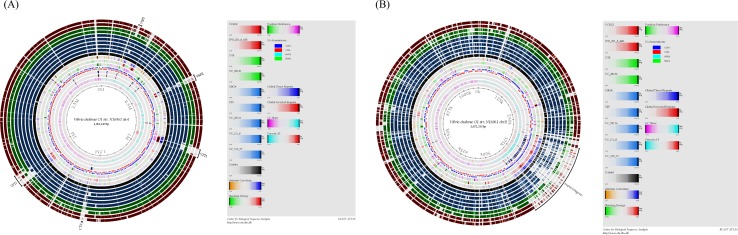
Genomic variation of representative *Vibrio cholerae* strains in Thailand. BLAST atlas with chromosome I (A) and II (B) of *V*. *cholerae* N16961 as reference strain (black) followed by the nine representative strains of serogroup O1, O139, and non-O1/non-O139 composed of serogroup O1 (blue) (clinical strains: 510/77, typical El Tor; VCO1-8, classical; 22116 and P25, El Tor variant; environmental strain: MK14, hybrid El Tor), O139 (green) (clinical strains: 22136, environmental strain: DT8), and non-O1/non-O139 (red) (clinical strains: IPD22I/8 44B, environmental strain: VCR12).

The distribution of virulence-associated genes and pathogenicity islands among the 78 *V*. *cholerae* strains was determined using MyDbFinder (Fig 1 and Table 1). All strains harbored the virulence-associated genes *hlyA*, *rtxA*, and *toxR*, with only the *stn* gene absent. Ten of the 17 virulence-associated genes (*ctxA*, *ctxB*, *zot*, *ace*, *tcpA*, *hlyA*, *mshA*, *rtxA*, *ompU*, and *toxR*) were found in 34 of the clinical strains (serogroup O1 and O139). Moreover, these strains contained the pathogenicity islands (PAIs) VPI-1, VPI-2, VSP-1, and VSP-2, except for two strains of O139 (6668/3 and 6225/3), which lacked VSP-2. All non-O1/non-O139 strains obtained from a clinical source harbored the *hlyA*, *rtxA*, and *toxR* genes, whereas strain IPD1231/8 52B in addition also harbored the *mshA*, TTSS, and VPI-2. Two out of four O1 strains of environmental origin harbored *hlyA*, *mshA*, *rtxA*, *ompU*, *toxR*, and VPI-2. Only one O1 strain contained the genes *chxA* (TC 22), TTSS (MK14), and VSP-1 (MK14). Among environmental strains, the virulence-associated genes and the PAIs of the non-O1/non-O139 similar to the O1 strain were detected but lacked TTSS and VSP-1. All four O139 strains harbored *hlyA*, *rtxA*, *ompU*, *toxR*, and VPI-2. Nine *V*. *cholerae* genomes based on the different serogroups, biotypes, and sources were compared using a BLAST atlas. The atlas revealed several variable genomic regions in chromosome I (Fig 2A) and II (Fig 2B). VPI-1, VPI-2, VSP-1, and VSP-2 were determined in the chromosome I among the regions of PAIs including CTXϕ, especially the clinical strains of O1 El Tor (510/77, 22116, P25), and O139 (22136). The O1 Classical (VC O1-8) and non-O1/non-O139 (IPD221/8 44B) strain lacked VSP-1 and VSP-2. Among the environmental strains, the O1 strain (MK14) harbored two PAIs, VSP-1 and VPI-2, while both O139 (DT8) and non-O1/non-O139 strains (VCR12) harbored only VPI-2. A large genomic island, super-integron, located in the chromosome II, showed more genetic diversity and obviously differed among these strains.

**Table 1:** Occurrence of virulence-associated genes among *Vibrio cholerae* strains from Thailand.

Serogroup	Source	No. (%) of strains											
		*ctxA*	*ctxB*	*zot*	*ace*	*tcpA*	*hlyA*	*mshA*	*rtxA*	*ompU*	*toxR*	*chxA*	TTSS^a^
**O1 **	**Clinical**	30	29	30	30	39	40	40	40	40	40	0	0
	**(n = 40)**	(75.0)	(72.5)	(75.0)	(75.0)	(97.5)	(100.0)	(100.0)	(100.0)	(100.0)	(100.0)		
	**Environmental**	0	0	0	0	0	4	3	4	2	4	1	1
	**(n = 4)**						(100.0)	(75.0)	(100.0)	(50.0)	(100.0)	(25.0)	(25.0)
**O139**	**Clinical**	11	6	9	11	12	12	12	12	12	12	0	0
	**(n = 12)**	(91.7)	(50.0)	(75.0)	(91.7)	(100.0)	(100.0)	(100.0)	(100.0)	(100.0)	(100.0)		
	**Environmental**	0	0	0	0	0	4	0	4	4	4	0	0
	**(n = 4)**						(100.0)		(100.0)	(100.0)	(100.0)		
**non-O1/non-O139**	**Clinical**	0	0	0	0	0	2	1	2	0	2	0	1
	**(n = 2)**						(100.0)	(50.0)	(100.0)		(100.0)		(50.0)
	**Environmental**	0	0	0	0	0	16	3	16	2	16	3	0
	**(n = 16)**						(100.0)	(18.8)	(100.0)	(12.5)	(100.0)	(18.8)	
**Total (n = 78) **		41	35	39	41	51	78	59	78	60	78	4	2
		(52.6)	(44.9)	(50.0)	(52.6)	(65.4)	(100.0)	(75.6)	(100.0)	(76.9)	(100.0)	(5.1)	(2.6)

All *V*. *cholerae* strains lacked *stn* gene.

^a^ Positive all four genes: *vcsC2*, *vcsN2*, *vcsV2*, and *vspD*

### Antimicrobial resistant strains, antimicrobial resistance genes, class 1 integron, and SXT element

The MIC determination of all 78 *V*. *cholerae* strains revealed that 48 of them originating between 1991 and 2013 were resistant to at least one antimicrobial (Table 2). The 48 strains were resistant to TMP (52.6%), SMX (48.7%), NAL (43.6%), TET (14.1%), AMP (7.7%), and AZM (6.4%). Moreover, 27 (56.3%) of the 48 antimicrobial resistant strains were considered multidrug resistant (MDR) and conferred resistance to three or more antimicrobial classes and exhibited four distinct MDR patterns: NAL-SMX-TMP, NAL-TET-TMP, NAL-SMX-TET-TMP, and AZM-NAL-SMX-TET-TMP (Table F in S1 File). It is noteworthy to mention that some resistance genes were observed among the strains being phenotypically susceptible. These strains were of O1, O139, and non-O1/non-O139, isolated between 1983 and 2010 and harbored the *cat*B9 (60.3%) and *flo*R (35.9%) conferring resistance to chloramphenicol (O-acetyltransferase activity) and florfenicol (co-resistance to both chloramphenicol and florfenicol), respectively.

**Table 2:** Frequency of resistance of *Vibrio cholerae* strains in Thailand.

Variable	Serogroup	No. of strains	No. (%) of resistant strains	No. (%) of strains resistant to various antimicrobial agents indicated CLSI clinical breakpoints values (μg/ml)					
				AMP	AZM	NAL	SMX	TET	TMP
				≥32	>2	≥32	≥512	≥16	≥4
**Year:**									
**1983–1990**	**O1**	7	0	0	0	0	0	0	0
	**non**	2	0	0	0	0	0	0	0
**1991–2000**	**O1**	3	0	0	0	0	0	0	0
	**O139**	12	12 (100.0)	0	0	0	12 (100.0)	0	12 (100.0)
	**non**	4	3 (75.0)	1 (25.0)	0	2 (50.0)	0	1 (25.0)	2 (50.0)
**2001–2010**	**O1**	32	27 (84.4)	0	5 (15.6)	27 (84.4)	26 (81.3)	10 (31.3)	27 (84.4)
	**non**	5	1 (20.0)	0	0	1 (20.0)	0	0	0
**2011–2013**	**O1**	2	0	0	0	0	0	0	0
	**O139**	4	4 (100.0)	4 (100.0)	0	4 (100.0)	0	0	0
	**non**	7	1 (14.3)	1 (14.3)	0	0	0	0	0
**Source:**									
**Clinical**		54	40 (74.1)	1 (1.9)	5 (9.3)	27 (50.0)	38 (70.4)	10 (18.5)	39 (72.2)
**Environmental**		24	8 (33.3)	5 (20.8)	0	7 (29.2)	0	1 (4.2)	2 (8.3)
**Total**		78	48 (61.5)	6 (7.7)	5 (6.4)	34 (43.6)	38 (48.7)	11 (14.1)	41 (52.6)

Abbreviations: AMP, ampicillin; AZM, azithromycin; NAL, nalidixic acid; SMX, sulfamethoxazole; TET, tetracycline; TMP, trimethoprim; non, non-O1/non-O139. No resistance observed for cefotaxime (R ≥4 μg/ml), ceftazidime (R ≥16 μg/ml), chloramphenicol (R ≥32 μg/ml), ciprofloxacin (R ≥4 μg/ml), gentamicin (R ≥16 μg/ml), meropenem (R ≥16 μg/ml), and tigecycline (R >2 μg/ml) (tigecycline was interpreted according to EUCAST based on clinical breakpoint.

**Fig 3:**
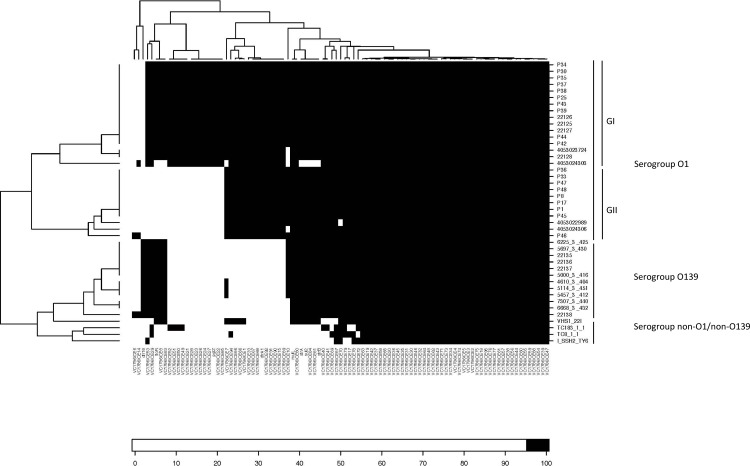
Genetic variation of SXT element in *Vibrio cholerae*. The SXT structures of among 43 *V*. *cholerae* strains from Thailand were compared. Reads were mapped to genes of ICE*Vc*Hai1 (accession no. JN648379) and *dfr*A18 gene in SXTMO10 element (accession no. AY034138).

The presence of the specific integrase genes of class 1 integron (*int*I gene) and SXT element (*int*_SXT_ gene) were *in silico* determined among the 78 *V*. *cholerae* strains using MyDbFinder (Table 3). All of the strains lacked the *int*I gene. In contrast, 43 strains of *V*. *cholerae* serogroups O1, O139, and non-O1/non-O139 isolated during 1991 to 2013 presented the *int*_SXT_. The SXT element harbored the following antimicrobial resistance genes: *sul*2, *dfr*A1, *dfr18*, *flo*R, *str*A, and *str*B, which are mostly associated with SMX and TMP resistant strains (Fig 3).

**Table 3:** Frequency of SXT element and antimicrobial resistance genes in *Vibrio cholerae* strains, Thailand.

Year	Serogroup	SXT	No. (%) of strains													
			Aminoglycoside		Beta-lactam			Phenicol		Sulphonamide	Trimethoprim		MLS	Quinolone		
			*str*A	*str*B	*bla*P1	*bla*CARB-7	*bla*CARB-9	*cat*B9	*flo*R	*sul*2	*dfr*A1	*dfr*A18	*mph*(A)	*qnr*VC5	GyrA(S83I)	ParC(S85L)
**1983–1990**	**O1**	0	0	0	0	0	0	2	0	0	0	0	0	0	0	0
								(28.6)								
	**non-O1/ non-O139**	0	0	0	0	0	0	0	0	0	0	0	0	1	0	0
														(50.0)		
**1991–2000**	**O1**	0	0	0	0	0	0	1	0	0	0	0	0	0	0	0
								(33.3)								
	**O139**	12	12	12	0	0	0	12	12	12	0	12	0	0	0	0
		(100.0)	(100.0)	(100.0)				(100.0)	(100.0)	(100.0)		(100.0)				
	**non-O1/non-O139**	1	1	1	1	0	1	2	1	1	0	0	0	0	2	2
		(25.0)	(25.0)	(25.0)	(25.0)		(25.0)	(50.0)	(25.0)	(25.0)					(50.0)	(50.0)
**2001–2010**	**O1**	27	26	26	0	0	0	28	15	26	27	0	6	0	27	27
		(84.4)	(81.3)	(81.3)				(87.5)	(46.9)	(81.3)	(84.4)		(18.8)		(84.4)	(84.4)
	**non-O1/non-O139**	2	0	0	0	0	0	2	0	0	0	0	0	0	1	1
		(40.0)						(40.0)							(20.0)	(10.0)
**2011–2013**	**O1**	0	0	0	0	0	0	0	0	0	0	0	0	0	0	0
	**O139**	0	0	0	4	0	4	0	0	0	0	0	0	0	4	4
					(100.0)		(100.0)								(100.0)	(100.0)
	**non-O1/non-O139**	1	0	0	0	1	0	0	0	0	0	0	0	0	0	0
		(14.3)				(14.3)										
**Total **		43	39	39	5	1	5	47	28	39	27	12	6	1	34	34
		(55.1)	(50.0)	(50.0)	(6.4)	(1.3)	(6.4)	(60.3)	(35.9)	(50.0)	(34.6)	(15.4)	(7.7)	(1.3)	(43.6)	(43.6)

Abbreviations: MLS, macrolide-lincosamide-streptogramin

The majority of *V*. *cholerae* strains (52.6%) were resistant to TMP, of which strains belonging to serogroup O1 (2001–2005) contained the *dfr*A1 gene and O139 strains (1991–2000) contained the *dfr*A18 gene (Table 3). All 38 (48.7%) strains conferring resistance to SMX contained *sul*2 gene. Among the six AMP-resistant strains (7.7%), four O139 environmental strains (DT8, DT7, WKB T9, and PKN T5) and one clinical non-O1/non-O139 strain (1231/8 52B) harbored the *bla*_P1_ and the *bla*_CARB-9_ gene, respectively, whereas only one non-O1/non-O139 environmental strain (I-WASTE-HSH1-TY2) harbored the *bla*_CARB-7_ gene. All five clinical O1 strains (6.4%) resistant to AZT contained the *mph*A gene. Interestingly, the 11 strains resistant to TET lacked resistance genes. The genes *str*A and *str*B conferring resistance to streptomycin were present in 39 strains (50%) including 12 strains (100%) of O139 and 1 strain (25%) of non-O1/non-O139 isolated during 1991–2000.

Amino acid substitutions in codon *gyr*A (Ser83Ile) and *par*C (Ser85Leu) were observed in 34 NAL-resistant strains (43.6%) isolated between 1991 and 2013 belonging to serogroup O1, O139, and non-O1/non-O139. In addition, one non-O1/non-O139 strain (1262 W278) conferred resistance to quinolone harboring the *qnr*VC5.

The whole genome sequence of the strains harboring the SXT element revealed a structure organized similar to ICE*Vch*Hai1 and SXT^MO10^ in the GenBank (Fig 3). Most strains except for 4053024303, 4053024306, and 22138 shared the similar structures of SXT element with common known deletions in loci VC1786ICE6 and VC1786ICE14. The variations in the SXT structures separated the individual serogroup O1, O139, and non-O1/non-O139 into distinct branches of the phylogenetic tree (Fig 3). The SXT elements of O1 strains were divided into two clades (GI and GII). The SXT structure of GI was highly similar to the structure of ICEVchHai1. Nineteen loci including *dfr*A18 and *flo*R were absent in GII. The SXT structures among the O139 strains harbored loci similar to SXT^MO10^ and ICEVchHai1 but lacked 25 loci including *dfr*A1. For non-O1/non-O139 strains, four strains harbored the SXT element and their SXT structures were similar to those of O139 strains. Only one resistant strain, VHS1-22I, harbored *flo*R, *str*A, *str*B, and *sul*2 genes. Two susceptible strains and one NAL-resistant strain did not contain these antimicrobial resistance genes including *dfr*A18 and *dfr*A1.

## Discussion

Since 1982, *V*. *cholerae* has been present and emerging in Thailand [40]. In the last decade, sporadic cholera cases have been observed in Thailand caused primarily by *V*. *cholerae* O1 and O139. In this study, we found that the phenotypic results characterizing *V*. *cholerae* were all in concordance with the *in silico* genotypic data revealed by WGS targeting the following genes: *ompW*, *rfbV*, *wbfZ*, *ctxB*, *rstR*, and *tcpA*. These genes have previously been used to classify *V*. *cholerae* strains [27, 30, 41–43]. The tested strains were classified into serogroups O1, O139, and non-O1/non-O139 showing that both *V*. *cholerae* serogroup O1 and O139 are present in Thailand and have potentially caused cholera.

In Thailand, several studies have reported the emergence of *V*. *cholerae* however, the biotype *V*. *cholerae* O1 classical has not been detected since 1986 [27, 44–46]. Interestingly, this study revealed that two strains obtained from stool samples in 2000 were identified as the Classical biotype and were genetically similar to the strains related to the 6^th^ cholera pandemic (Table E in S1 File). This indicated that the Classical biotype might have re-emerged, causing cholera outbreaks in Thailand after having been absent for several years during the 6^th^ cholera pandemic. The decline of typical El Tor strains coincided with the first reports from Bangladesh of the emergence of the El Tor variant strain [16]. Furthermore, the El Tor variants possessing both the Classical and El Tor biotypes were recovered from clinical strains during 2004–2010. Detection of the El Tor variant was previously reported in Cameroon, India, and Thailand [18, 32, 41]. The variant of the Classical and El Tor biotypes increases the severity of the disease and may result in higher morbidity and mortality [47, 48]. Kim *et al*. suggested that the El Tor variant possessing the Classical biotype originated through recombination between the Classical and El Tor types of CTXϕ [49]. One hybrid strain of this study, MK14, originating from a river water sample, lacked the biotype-specific genes as well as the main virulence genes (*ctxAB* and *tcpA*), suggesting it to be a non-toxigenic strain and in agreement with previous reports [50, 51]. The non-toxigenic strains, however, have been responsible for causing mild to moderate diarrhea in human volunteers in clinical trials [2]. These El Tor variant strains clustered together with the clinical strains including typical El Tor biotype and O139 serogroup. Moreover, the *in silico* MLST analysis showed that the clinical strains had a highly genetic relationship with the pandemic and outbreak strains. The majority of the clinical strains O1 and O139 belonged to ST69 and showed genetic similarity to the 7^th^ pandemic strain (N16961), the Haitian outbreak strain (2010EL-1786), and the Cameroon outbreak strains [32]. In addition, all of the clinical strains harbor the specific gene marker of the 7^th^ pandemic clone. These findings suggest that the clinical strains (1983–2010) in Thailand might originate from a common ancestor of the 7^th^ pandemic strain. The STs of the clinical strains showed that they were closely related to the pre 6^th^ pandemic strain (M66-2) and a previous outbreak strain in Thailand (MS6) [52]. The clinical strains of O1 and O139 were highly conserved with regard to MLST (ST69) but contained different virulence genes, particularly *ctxAB* and *tcpA*. These findings have previously been reported and might be a result of horizontal gene transfer [36, 53, 54]. The *in silico* MLST analysis clearly showed discrimination amongst the different sources (clinical and environmental) and serogroups O1 and O139 as compared with non-O1/non-O139 strains. The clinical strains of O1 and O139 were highly conserved with regard to MLST (ST69), while the environmental strains of O1, O139, and non-O1/non-O139 and the clinical strains of non-O1/non-O139 revealed different and novel STs. This indicates that the environmental strains including non-O1/non-O139 were highly diverse; however, these results might be caused by gene recombination and/or mutation [36].

Furthermore, the environmental strains could be distinguished from the clinical strains using *in silico* MLST based on the difference in the virulence gene profiles. The environmental strains of O1 and O139 lacked the CTXϕ and *tcpA* genes, especially. However, these strains harbored other virulence genes similar to non-O1/non-O139. Both *chxA* and TTSS genes were frequently found among non-O1/non-O139 pathogenic strains and associated with diarrhea [36, 51, 55]. However, the environmental O1 strains in this study harbored *chxA* gene (TC22) and TTSS (MK14), indicating virulence potential to cause disease.

Our study showed that the antimicrobial resistance profiles SMX-TMP and NAL-SMX-TMP were predominant among the clinical strains of serogroup O139 and O1, respectively. In addition, other clinical strains exhibited resistance to TET, AZM, and AMP in contrast to the environmental strains which were mostly resistant to NAL followed by AMP, TMP, and TET. Previous reports have described different antimicrobial resistant profiles compared with those from Thailand, such as resistance to furazolidone, NAL, sulfisoxazole, streptomycin, and trimethoprim/sulfamethoxazole in Haiti [56] as well as TET, streptomycin, sulfisoxazole and trimethoprim in China [57]. During 2003–2011, *V*. *cholerae* O1 has been reported as being resistant to erythromycin, TET, trimethoprim/sulfamethoxazole, and AMP in Thailand [27].

Our study showed a similar concordance between the antimicrobial susceptibility testing data and the *in silico-*detected corresponding resistance genes in the *V*. *cholerae* strains using the ResFinder bioinformatics tool [34]. A few disagreements were observed and confirmed by re-testing the MIC determination. These discrepancies related to TET-resistant strains in which no conferring resistance genes or other resistance mechanisms could be detected. This phenomenon is well-known and has previously been reported related to potential efflux pumps [58]. In contrast, we observed some strains that harbored both *flo*R and *cat*B9 but displayed a susceptible phenotypic resistance profile. This observation has also been described in a recent publication describing the cholera in Haiti [56]. Similarly, susceptible non-O1/non-O139 strains harboring the *qnrVC5* gene did not express resistance to quinolone. Normally, one would anticipate isolates that harbor the genes *flo*R and *cat*B9 would be associated with reduced susceptibility to CHL [59] and those that harbor the gene *qnrVC5* would be associated with quinolone resistance. These abnormalities are most likely linked to incorrect interpretative criterion.

According to World Health Organization (WHO) recommendations, TET and CIP are the drugs of choice for the treatment of cholera. Unfortunately, there is a lack of prudent usage in Thailand because these antimicrobials are being misused/overused in the agricultural section [60]. During 2003–2011, the endemic cholera strains in Thailand were resistant to TET, whereas cholera was still susceptible to CIP as proven by Chomvarin *et al*., 2013 [27] and in this study. Amino acid substitutions in *gyr*A and *par*C are the main mechanism responsible for quinolone resistance in *V*. *cholerae* [56, 58, 61]. In this study, the same point mutations in *gyr*A (S83I) and *par*C (S85L) were detected among NAL-resistant strains found in both clinical and environmental sources.

The SXT element is an ICE that translocates a panel of antimicrobial resistance genes via horizontal gene transfer [62]. The first SXT, SXT^MO10^, was discovered in *V*. *cholerae* O139 strain MO10. It harbored resistant determinants to trimethoprim (*dfr*A18), streptomycin (*str*A, *str*B), sulfamethoxazole (*sul*2), and chloramphenicol (*flo*R) [63]. Other ICEs identified in O1 and non-O1/non-O139 harbor a similar set of resistance genes as the SXT^MO10^ strain [28, 64]. Recently, WGS has been used to identify a variant of SXT in a Haitian O1 strain, ICE*Vch*Hai1 harboring *dfr*A1, *str*A, *str*B, *sul*2, and *flo*R [56]. We analyzed the genetic variation in SXT elements by comparing with gene loci in ICE*Vch*Hai1 and *dfr*A18 in SXT^MO10^. ICE*Vch*Hai1 has previously been used as the reference for comparison with the SXT element in India [64]. In this study, we found that the SXT in each of the different serogroups O1, O139, and non-O1/non-O139 were distinctly different. The SXT structures of the O1 strains showed a higher genetic similarity with ICE*Vch*Hai1 than the SXT structures of O139 and non-O1/nonO139 strains. This indicated that the acquired SXT element in the O1 Thai strains were similar to those of the Haitian and Indian strains. These findings are consistent with a previous study that showed identity of SXT within the same serogroup of *V*. *cholerae* [28].

In this study, we found that the re-occurrence of classical toxigenic strains have been persisted since 2000 in Thailand. The variation of phenotypic and genotypic characteristics shows that the *V*. *cholerae* O1 biotype El Tor variant has caused the majority of the outbreaks since 2004. The *V*. *cholerae* O1 and O139 obtained from clinical source commonly harboured CTXϕ and *tcp*A. Conversely, their environmental strains lacking those virulence genes could be detected. Moreover, the occurrence of SXT element and resistance genes conferring antimicrobial resistance was encountered among Thai strains. These findings suggest that lysogenicity of *V*. *cholerae* O1 for CTXϕ and other genetic markers including resistance genes should be further intensively surveillance and control. Future application of WGS combined with bioinformatic tools, such as MLST [35], MyDbFinder, ResFinder [34], and VcTypeFinder (in development), have in this study proven the power and are highly discriminatory methods in understanding the epidemiology of *V*. *cholerae*.

## Conclusions

In this study, we used WGS and bioinformatic tools to analyze both historical and contemporary *V*. *cholerae* circulating in Thailand. To our knowledge, this is the first time since 1986 that the presence of *V*. *cholerae* O1 classical has been reported causing cholera outbreaks in Thailand. We found that the majority of the pathogenic strains belonged to *V*. *cholerae* O1 El Tor variant and O139. *In silico* analysis showed that the clinical strains shared common genetic background as well as harbored virulence genes, PAIs and mobile genetic elements associated with antimicrobial resistance while environmental strains were highly diverse. This study contributed to understanding the epidemiology of *V*. *cholerae* in Thailand that ultimately can be applied for control measures and management of the disease in Thailand.

## Supporting Information

S1 File Supplementary_table1-Sequence_info. (XLS)
